# Cerebral Blood Flow in Low Intracranial Pressure Headaches—What Is Known?

**DOI:** 10.3390/brainsci10010002

**Published:** 2019-12-19

**Authors:** Magdalena Nowaczewska, Henryk Kaźmierczak

**Affiliations:** 1 Department of Pathophysiology of Hearing and Balance System, Faculty of Medicine, Collegium Medicum in Bydgoszcz, Nicolaus Copernicus University, M. Curie 9, 85-090 Bydgoszcz, Poland; 2 Department of Otolaryngology, Head and Neck Surgery, and Laryngological Oncology, Ludwik Rydygier Collegium Medicum in Bydgoszcz Nicolaus Copernicus University, M. Curie 9, 85-090 Bydgoszcz, Poland

**Keywords:** cerebral blood flow, intracranial pressure, intracranial hypotension, orthostatic headaches, post-dural-puncture headaches, transcranial doppler, cerebrospinal fluid, cerebral autoregulation

## Abstract

Headaches attributed to low cerebrospinal fluid (CSF) pressure are described as orthostatic headaches caused by spontaneous or secondary low CSF pressure or CSF leakages. Regardless of the cause, CFS leaks may lead to intracranial hypotension (IH) and influence cerebral blood flow (CBF). When CSF volume decreases, a compensative increase in intracranial blood volume and cerebral vasodilatation occurs. Sinking of the brain and traction on pain-sensitive structures are thought to be the causes of orthostatic headaches. Although there are many studies concerning CBF during intracranial hypertension, little is known about CBF characteristics during low intracranial pressure. The aim of this review is to examine the relationship between CBF, CSF, and intracranial pressure in headaches assigned to low CSF pressure.

## 1. Introduction

According to the International Headache Society, headaches attributed to low cerebrospinal fluid (CSF) pressure are described as orthostatic headaches caused by spontaneous or secondary low CSF pressure or CSF leakages, usually accompanied by neck pain, tinnitus, changes in hearing, photophobia, and/or nausea. These symptoms remit after normalization of CSF pressure or successful sealing of the CSF leak [1]. Due to several possible causes of headaches, they can be divided into categories: post-dural-puncture headaches (PDPH), occurring within five days of a lumbar puncture (LP); CSF fistula headaches, occurring after a procedure or trauma causing persistent cerebrospinal fluid leakage resulting in low intracranial pressure; and headaches attributed to spontaneous intracranial hypotension (SIH), caused by low CSF pressure of spontaneous origins [1]. Such a leakage of CSF most commonly occurs after dural punctures for a diagnostic LP, myelography, or spinal anesthesia. Other rare causes of CSF volume depletion include total body water loss (true hypovolemic state), various types of trauma, and CSF shunt overdrainage [2]. Regardless of the cause, CFS leaks or depletion leads to intracranial hypotension (IH). Any change in intracranial pressure (ICP) may influence cerebral blood flow (CBF) [3,4,5]. Although there is plenty of data regarding CBF during intracranial hypertension, surprisingly little is known about CBF characteristics during low intracranial pressure. The objective of this paper is to present a review with the aim of establishing the relationship between CBF, CSF, and ICP in headaches attributed to low CSF pressure.

## 2. Cerebral Blood Flow and Intracranial Pressure

CBF is commonly defined as the volume of blood delivered to the brain tissue per minute, while ICP is defined as the pressure inside the skull and is the result of the interaction between the brain, CSF, and cerebral blood volume. Cerebral autoregulation (CA) is the mechanism responsible for maintaining constant CBF over a wide range of arterial blood pressures. CBF regulation is possible through neurogenic, myogenic, metabolic, or endothelial control to support CBF at an appropriate level for the brain’s metabolic oxygen requirements [6]. However, when the upper or lower limits of these autoregulatory mechanisms are exceeded, CBF becomes absolutely dependent on mean arterial pressure (MAP) [7]. Under normal, physiological conditions, the graphic recording of ICP is stable and regular, and ICP depends on CSF production volume, system resistance to reabsorption of CSF, and venous pressure in the intracranial space [7]. Another important variable is compliance, defined as the change in volume in response to a change in pressure. In a highly compliant system, changes in pressure are compensated and do not result in a direct change in volume [8]. Some authors have shown compromised cerebral autoregulation in patients with normal ICP, while others have reported CA impairments together with intracranial hypertension, probably secondary to vasomotor response impairment, blood–brain barrier disruption, and brain oedema [9,10]. Nevertheless, there is a lack of agreement in the literature regarding the relationship between damaged CA and ICP. In a review, de-Lima-Oliveira et al. found a correlation between intracranial hypertension and compromised CA [4]. They concluded that impaired CA may produce intracranial hypertension, but high ICP can also influence CA. Nevertheless, CA impairment is also observed in patients with normal ICP [4,11]. Sudden increases in ICP decrease cerebral perfusion pressure (CPP—the difference between the MAP and ICP), and thus can limit perfusion to the brain. This decrease in CPP can be partially compensated for by active cerebral vasodilatation to maintain CBF independently from decreases in CPP [5]. When CPP decreases, the cerebrovascular resistance (CVR) is reduced, followed by a decreasing of the blood flow velocity. Further increases of ICP can cause incremental CBF autoregulation to be lost. Compressed venous ends of the cerebral vessels may increase CVR and prevent the blood from returning. Subsequently, both the CBF and blood flow velocity gradually decrease. Persistent high ICP may reduce intracranial compliance, causing a further decline in CBF autoregulation [12]. Another very important parameter is critical closing pressure (CCP), described as the arterial blood pressure at which brain vessels collapse and CBF ceases. CCP is a valuable and clinically relevant tool, allowing estimate changes in the cerebrovascular tone and minimal CPP to prevent collapsing of vessels and ischemia [13,14]. Two compensatory adaptation mechanisms to raised ICP exist: when ICP moderately increases, CBF is preserved by decreases in vascular wall tension, while at higher levels of ICP, CPP is protected by the Cushing vasopressor response [15]. It was found that, with an increase in ICP, the middle cerebral artery (MCA) blood flow progressively decreases [12].

## 3. Monroe–Kellie Doctrine and Intracranial Hypotension Headaches

**Figure 1:**
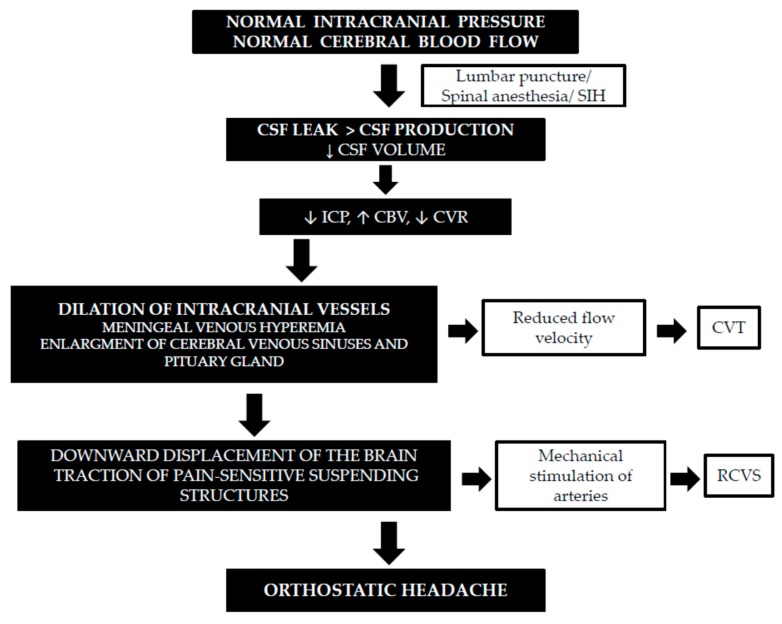
Cerebral blood flow characteristics in low intracranial pressure headaches [2,18,20,23,24,25,26,27,28,29,30,31]. Definitions: (SIH) spontaneous intracranial hypotension, (CSF) cerebrospinal fluid, (CBF) cerebral blood flow, (CVR) cerebrovascular resistance, (ICP) intracranial pressure, (CVT) cerebral venous thrombosis, and (RCVS) reversible cerebral vasoconstriction syndrome.

The Monroe–Kellie doctrine describes the following principle: “with intact skull, sum of volume of brain plus volume of CSF plus volume of intracranial blood is constant, and therefore decrease or increase in one will result in increase or decrease in one or both of the remaining two” [16,17]. In IH, CSF leaks through a known or probable meningeal hole at a rate that exceeds CSF production, leading to a decrease in CSF volume. Because brain volume has almost no capacity to expand, the intracranial blood volume has to compensate for the CSF volume loss [18,19]. It is known that veins are more easily expansible than arteries, and most of this compensation occurs through dilation of the veins because of their greater compliance [20]. This has been proven by magnetic resonance imaging (MRI) findings. The characteristic abnormalities identified in IH consist of the downward displacement of the brain, subdural fluid collections, distention of the cerebral and spinal venous systems, pachymeningeal enhancement, and expansion of the pituitary gland. The meningeal venous flow increase leads to diffuse pachymeningeal enhancement, as well as enlargement of the cerebral venous sinuses and pituitary gland as part of the compensatory hyperemia [2,16]. The main cause of orthostatic headaches in CSF leaks is descent of the brain and traction on its pain-sensitive structures. Sinking of the brain is exaggerated in the upright posture, which is responsible for the orthostatic nature of these headaches. When CSF volume decreases, in accordance with the Monroe–Kellie rule, there is cerebral vasodilatation as a result of an increase in blood volume, which may activate the trigeminovascular system and produce headaches, such as during migraine attacks. Because vasodilatation occurs in pain-sensitive blood vessels, it may also result in pain [2,21,22,23]. The cause of neck pain and radicular symptoms of the upper limbs, often described in IH patients, may be from traction on spinal and cranial nerves [23] (Figure 1).

## 4. Techniques to Measure Cerebral Blood Flow

Multiple methods have been developed to accurately measure CBF metabolism. Each method has its unique advantages, as well as limitations [32]. Current techniques to measure CBF can be invasive, requiring surgical access, catheterization, or arterial puncturing, whereas minimally invasive techniques require intravenous injection of a contrast agent [33]. The perfect and ideal imaging technique that allows continuous, noninvasive measurements of CBF and metabolism across the whole brain does not exist; no current imaging method meets all these criteria [34]. The accurate estimation of CBF is done by invasive methods; it requires complex and expensive equipment and cannot be used bedside. Most of these techniques offer snapshots of the CBF. MRI and PET may also provide some information about the metabolic state of the brain and quantitative measurements of CBF; however, those methods are costly and limited. Most invasive methods, except mobile Xenon-CT, require the transferring of patients to the radiological department [34]. One noninvasive, simple method of CBF assessment is the transcranial doppler (TCD). Although the TCD does not measure the CBF directly, only the flow velocity, which is a surrogate marker of CBF, it is widely used in clinical studies, mostly because it is relatively inexpensive, portable, and repeatable [35]. The TCD with a 2-MHz-pulsed doppler probe allows noninvasive, intermittent, or continuous assessments of the velocity of blood flow through large cerebral vessels. Measured indices of flow include systolic, mean, and diastolic flow velocities (Vs, Vm, and Vd) and the pulsatility index (PI). The PI is an indirect indicator of ICP changes. The value of the PI has traditionally been interpreted as a descriptor of distal cerebrovascular resistance. Some studies suggest a linear relationship between changes in the PI and ICP. A decrease in the PI may result both from lowered ICP and from vascular dilatation [36,37,38]. Although, it is worth noting that a correlation between the PI and ICP has been found in the context of intracranial hypertension (after traumas, intracranial hemorrhages, cerebral mass lesions, and hydrocephalus) and is strongest when ICP is over 20 mm [39,40]. Moreover, a change in CVR does not always influence blood flow velocity, because velocity is measured in big arteries while CVR is controlled by small arteries. Additionally, the PI is not dependent solely on CVR but is a result of the interaction between the CPP, the pulse amplitude of arterial pressure, cerebrovascular resistance, and compliance of the cerebral arterial bed [41]. However, the TCD technique is operator-dependent, and the values are affected by various local and systemic factors [35]. The TCD only measures CBF velocity and not CBF, per se. For example, there are differences between the TCD measure of blood flow velocity in MCAs and tissue-level CBF using arterial spin-labeling MRIs. CBF, as the volumetric flow of blood to an absolute mass of brain tissue, is defined as the product of a vessel cross-sectional area and the velocity of moving blood, with tissue mass being a function of density and volume. So, the TCD measurements of blood flow velocity can be used as an accurate surrogate for CBF only if both tissue weight and vessel area remains constant between individuals [42].

## 5. Cerebral Blood Flow in Intracranial Hypotension—Clinical and Experimental Studies 

See Table 1.

### 5.1. Transcranial Doppler Studies

In a study of 66 patients who required diagnostic lumbar punctures, CBF was assessed 24 h before and within 24 h after the procedures [28]. In patients who developed PDPHs, the post-puncture values of Vm in both MCAs were significantly lower than the parameters before punctures. The decrease of Vm observed in this group of patients may result from the dilatation of intracranial vessels, a consequence of lowered ICP, so the findings of this study partially confirmed the theory behind the etiology of PDPHs [28]. Another study aimed to check whether hydration before puncture influenced the incidences of post-dural-puncture headaches and affected CBF in 99 patients enrolled for diagnostic lumbar punctures [43]. The authors noticed that Vm values in both MCAs after punctures were significantly lower than respective baseline parameters in all patients, while the PI after punctures increased only in the PDPHs group. The large decrease in Vm in the PDPHs group probably caused compensatory vasoconstriction in the microcirculation, which resulted in the PI growth. It is worth noting that in the group of hydrated patients, PDPHs were significantly less frequent than in the group without hydration, and post-puncture Vm values were higher than in the group without hydration. This was probably why these patients less frequently developed the decrease of ICP values and PDPHs [43]. Similarly, Gobel et al. demonstrated a significant reduction in the mean flow velocity of the right MCA 48 h after lumbar punctures only in patients who developed PDPHs [44]. An analogous study was done with different groups of patients. Mowafy et al. studied the CBF during post-dural-puncture headaches in parturient patients undergoing elective cesarean sections, by means of the TCD. Contrary to previous studies, they noticed significant increases of Vm values in MCAs in all patients within the first 48 h after cesarean sections, as compared with those before delivery. In the PDPHs group, there was highly statistically significant decreases in the PI values at 24 h and 48 h, compared to the pre-puncture values. The explanation of these different results may be connected with pregnancy and delivery. After delivery, velocity progressively and rapidly increases in the first postpartum week, but this may be due to blood loss during delivery. The mechanism for these increases in the Vm might be also mild vasoconstriction, resulting from a sudden decrease in estrogen levels after delivery [45]. Only one doppler study examined intracranial veins. Chen et al. hypothesized that the superior ophthalmic vein might reflect the engorgement of the intracranial venous sinuses that occurs during IH, because it is a tributary of the cavernous sinus. They showed increased diameters and maximum flow velocities of the superior ophthalmic veins in patients with IH by means of transorbital color doppler flow imaging [46].

### 5.2. Different Techniques Studies

Pomeranz et al. measured regional CBF utilizing the hydrogen clearance method in the cerebral cortex and subcortical nuclei in 11 cats with stable blood pressures and intracranial hypotension of at least 15 torr. They noticed that regional CBF was unchanged relative to the baseline, and concluded that cerebral vascular autoregulation is maintained during significantly increased perfusion pressure due to negative intracranial pressure. Additionally, the symptomatology of clinical intracranial hypotension is not due to decreased cerebral perfusions [29]. Contrary to these results, Salmon et al., in a study with dogs, found that the average increase in cortical blood flow was 30 mL/100 g/min (47%) when the intracranial pressure was lowered acutely from 100 mm to 40 mm of CSF [47]. In this study, CBF was determined using the inert radioactive gas 133Xe. Using a ventriculoatrial shunt, they also produced permanent intracranial hypotension in seven demented patients. The mean post shunt pressure was 50 mm of CSF and decreased the cerebral vascular resistance by 32%, increased the cortical blood flow by 37%, and increased the relative weight of functional grey matter by 44%. They concluded that an increase in the pressure differentials between the precapillary arterioles and the veins probably increased CBF and dilated vessels in response to decreased external pressure. Thus, increases in CBF may be the mechanism by which patients with normal pressure hydrocephalus who are shunted improve [47]. Honig et al. assessed the manifestations of cerebral venous thrombosis (CVT) associated with IH following lumbar punctures or spinal anesthesia [26]. IH was diagnosed in 11/42 (26%) of CVT patients. They demonstrated that patients presenting with CVT associated with IH had a more rapid and severe course of illness, higher rates of venous infraction, and symptomatic seizures, compared with CVT patients not associated with IH. CVT may result from enlargement of venous spaces, which reduces flow velocity in intracranial veins. This slow flow secondary to venous vasodilatation may be further aggravated by impaired venous drainage in the recumbent position after lumbar punctures. Stretching of the cerebral vessels may lead to traumatic damage of the venous endothelial and contribute to CVT and subdural collection formation. Moreover, CVT formation may be aggravated by hypercoagulopathy [26]. Schmidt et al. measured cerebral hemodynamic changes induced by a lumbar puncture in good-grade subarachnoid hemorrhages (SAH). Regional CBF was measured semi-quantitatively with O 15 PET before and after LPs. They demonstrated a heterogeneous and biphasic change in cerebral hemodynamics. Regional CBF was not kept constant and either augmented or decreased after the drop in ICP, so cerebrovascular reactivity was spatially heterogeneous within the brain [48]. The authors presented a different approach that ICP drop is a pure biomechanical trigger. A 20 ml LP seemed to increase blood flow in certain regions of the brain tissue but also to produce a reduction of the blood delivery in various regions, such as in brain tissue remote from the bleed [48].

### 5.3. Case Reports

Chaves et al. reported a patient who developed multiple cerebral infarcts from reversible cerebral vasoconstriction (RCVS) after intracranial hypotension caused by an iatrogenic lumbar CSF leak. These authors hypothesized that the cerebral vasoconstriction was caused by the sinking of the brain and associated structures, which was caused by CSF loss [49]. Mechanical stimuli—along with chemical, neurogenic, and electrical factors—can provoke cerebral arterial constriction. RCVS also has been reported during the postpartum period, which may be associated with dural puncture and CSF loss. Feil et al. reported a case of RCVS and posterior reversible encephalopathy syndrome associated with intracranial hypotension in a young woman 6 days post peridual anesthesia for a cesarean section [25]. Schieving et al. described a unique patient with spontaneous intracranial hypotension who developed severe but transient segmental stenosis of large and medium-sized cerebral arteries [30]. Some studies confirmed dilation of the venous side of the circulation in IH. Cerebral angiography showed an enlarged anterior falx artery and markedly enlarged cortical and medullary veins in one case of SIH [27].

## 6. Conclusions

Despite a small number of studies, there is some evidence indicating CBF changes in low intracranial pressure headaches, but the results are incoherent. Most studies revealed the dilatation of intracranial vessels, especially veins with CBF increases and flow velocity decreases, whereas some data showed IH-related vasoconstrictions and flow velocity increases. These ambiguous results may have arisen from different methods used for CBF assessments or heterogeneous groups of patients. Although there is a link between low intracranial pressure and cerebral blood flow, a larger study should be performed to assess possible connections between intracranial pressure, CBF, CSF, vein and artery flow, CVR, and cerebral autoregulation in low intracranial pressure headaches.

## Figures and Tables

**Table 1:** Studies of CBF in IH. Definitions: (LP) lumbar puncture, (TCD) transcranial doppler, (Vm) mean velocity, (Vmax) maximum velocity, (MCA) middle cerebral artery, (PDPH) post-dural-puncture headache, (IH) intracranial hypotension, (CBF) cerebral blood flow, (CSF) cerebrospinal fluid, (CVR) cerebrovascular resistance, (ICP) intracranial pressure, (Xe) xenon, (CDFI) color doppler flow imaging, (PET) positron emission tomography, (PI) pulsatility index, and (SAH) subarachnoid hemorrhage.

Author (Year)	Study Group (Number of Participants)	Technique to Measure CBF	Results
Nowaczewska (2012) [28]	Patients before and after LP (*n* = 66)	TCD	Vm decreased in MCAs 24 h after LP in PDPH group.
Nowaczewska (2019) [43]	Patients before and after LP (*n* = 99)	TCD	Vm decreased in MCAs after LP in all patients. PI increased after LP only in the PDPH group.
Gobel (1990) [44]	Patients before and after LP (*n* = 45)	TCD	Vm decreased in right MCA 48 h after LP in PDPH group.
Mowafy (2019) [45]	Parturient patients undergoing elective cesarean sections (*n* = 90)	TCD	Vm increased in MCAs in all patients within the first 48 h after cesarean sections. PI decreased at 24 h and 48 h in PDPH group.
Chen (1999) [46]	IH patients (*n* = 25)	CDFI	Vmax and diameter of the superior ophthalmic veins increased in patients with IH.
Pomeranz (1993) [29]	IH cats (*n* = 11)	hydrogen clearance method	Regional CBF was unchanged relative to the baseline.
Salmon (1971) [47]	IH dogs (*n* = 7)	radioactive gas 133Xe	Increase in cortical blood flow when ICP was lowered acutely from 100 mm to 40 mm of CSF.
Salmon (1971) [47]	IH patients (*n* = 7)	radioactive gas 133Xe	After lowering CSF pressure, CVR decreased, the cortical blood flow increased, and the relative weight of functional grey matter increased.
Schmidt (2012) [48]	SAH patients before and after LP (*n* = 6)	O 15 PET	Heterogeneous and biphasic changes in cerebral hemodynamics. Regional CBF was not kept constant and either augmented or decreased after the drop in ICP.

## References

[1] Headache Classification Committee of the International Headache Society (IHS) (2018). The International Classification of Headache Disorders, 3rd edition. Cephalalgia.

[2] Mokri B. (2013). Spontaneous low pressure, low CSF volume headaches: Spontaneous CSF leaks. Headache.

[3] Cipolla M.J. (2009). The Cerebral Circulation.

[4] de-Lima-Oliveira M., Salinet A.S.M., Nogueira R.C., de Azevedo D.S., Paiva W.S., Teixeira M.J., Bor-Seng-Shu E. (2018). Intracranial Hypertension and Cerebral Autoregulation: A Systematic Review and Meta-Analysis. World Neurosurg..

[5] Donnelly J., Czosnyka M., Harland S., Varsos G.V., Cardim D., Robba C., Liu X., Ainslie P.N., Smielewski P. (2017). Cerebral haemodynamics during experimental intracranial hypertension. J. Cereb. Blood Flow Metab..

[6] Armstead W.M. (2016). Cerebral Blood Flow Autoregulation and Dysautoregulation. Anesthesiol. Clin..

[7] Rodríguez-Boto G., Rivero-Garvía M., Gutiérrez-González R., Márquez-Rivas J. (2015). Basic concepts about brain pathophysiology and intracranial pressure monitoring. Neurologia.

[8] Varsos G.V., Kasprowicz M., Smielewski P., Czosnyka M. (2014). Model-based indices describing cerebrovascular dynamics. Neurocrit. Care.

[9] Czosnyka M., Smielewski P., Piechnik S., Steiner L.A., Pickard J.D. (2001). Cerebral autoregulation following head injury. J. Neurosurg..

[10] Cremer O.L., van Dijk G.W., Amelink G.J., de Smet A.M., Moons K.G., Kalkman C.J. (2004). Cerebral hemodynamic responses to blood pressure manipulation in severely head-injured patients in the presence or absence of intracranial hypertension. Anesth. Analg..

[11] Puppo C., Camacho J., Varsos G.V., Yelicich B., Gómez H., Moraes L., Biestro A., Czosnyka M. (2016). Cerebral Critical Closing Pressure: Is the Multiparameter Model Better Suited to Estimate Physiology of Cerebral Hemodynamics?. Neurocrit. Care.

[12] Wang Y., Duan Y.Y., Zhou H.Y., Yuan L.J., Zhang L., Wang W., Li L.H., Li L. (2014). Middle cerebral arterial flow changes on transcranial color and spectral Doppler sonography in patients with increased intracranial pressure. J. Ultrasound Med..

[13] Varsos G.V., Kolias A.G., Smielewski P., Brady K.M., Varsos V.G., Hutchinson P.J., Pickard J.D., Czosnyka M. (2015). A noninvasive estimation of cerebral perfusion pressure using critical closing pressure. J. Neurosurg..

[14] Varsos G.V., Richards H., Kasprowicz M., Budohoski K.P., Brady K.M., Reinhard M., Avolio A., Smielewski P., Pickard J.D., Czosnyka M. (2013). Critical closing pressure determined with a model of cerebrovascular impedance. J. Cereb. Blood Flow Metab..

[15] Donnelly J., Czosnyka M., Harland S., Varsos G.V., Cardim D., Robba C., Liu X., Ainslie P.N., Smielewski P. (2018). Increased ICP and Its Cerebral Haemodynamic Sequelae. Acta Neurochir. Suppl..

[16] Mokri B. (2001). The Monro-Kellie hypothesis: Applications in CSF volume depletion. Neurology.

[17] Wilson M.H. (2016). Monro-Kellie 2.0: The dynamic vascular and venous pathophysiological components of intracranial pressure. J. Cereb. Blood Flow Metab..

[18] Huang Y.M., Davidsson L. (2013). Sagging brain development after lumbar puncture agrees with Monro-Kellie hypothesis. J. Neurol..

[19] Karakis I., Nuccio A.H., Amadio J.P., Fountain A.J. (2017). The Monro-Kellie Doctrine in Action: Posterior Reversible Leukoencephalopathy Syndrome Caused by Intracranial Hypotension from Lumboperitoneal Shunt Placement. World Neurosurg..

[20] Fishman R.A., Dillon W.P. (1993). Dural enhancement and cerebral displacement secondary to intracranial hypotension. Neurology.

[21] Amorim J.A., de Barros M.V.G., Valença M.M. (2012). Post-dural (post-lumbar) puncture headache: Risk factors and clinical features. Cephalalgia.

[22] Lipman I.J. (1977). Primary intracranial hypotension: The syndrome of spontaneous low cerebospinal fluid pressure with traction headache. Dis. Nerv. Syst..

[23] Paldino M., Mogilner A.Y., Tenner M.S. (2003). Intracranial hypotension syndrome: A comprehensive review. Neurosurg. Focus.

[24] Abdel-Aziz S., Benzon H.T., Hurley R. (2018). Postmeningeal Puncture Headache and Spontaneous Intracranial Hypotension. Essentials of Pain Medicine.

[25] Feil K., Forbrig R., Thaler F.S., Conrad J., Heck S., Dorn F., Pfister H.W., Straube A. (2017). Reversible cerebral vasoconstriction syndrome and posterior reversible encephalopathy syndrome associated with intracranial hypotension. Neurocrit. Care.

[26] Honig A., Eliahou R., Pikkel Y.Y., Leker R.R. (2016). Iatrogenic intracranial hypotension and cerebral venous thrombosis. J. Neurol. Sci..

[27] Koss S.A., Ulmer J.L., Hacein-Bey L. (2003). Angiographic features of spontaneous intracranial hypotension. AJNR Am. J. Neuroradiol..

[28] Nowaczewska M., Książkiewicz B. (2012). Cerebral blood flow characteristics in patients with post-lumbar puncture headache. J. Neurol..

[29] Pomeranz S., Beni L., Shalit M.N. (1993). The effect of intracranial hypotension on cerebral blood flow in a feline model. Acta Neurochir. (Wien).

[30] Schievink W.I., Maya M.M., Chow W., Louy C. (2007). Reversible cerebral vasoconstriction in spontaneous intracranial hypotension. Headache.

[31] Yoon K.W., Cho M.K., Kim Y.J., Lee S.K. (2011). Sinus thrombosis in a patient with intracranial hypotension: A suggested hypothesis of venous stasis. a case report. Interv. Neuroradiol..

[32] Sudikoff S., Banasiak K. (1998). Techniques for measuring cerebral blood flow in children. Curr. Opin. Pediatr..

[33] Fantini S., Sassaroli A., Tgavalekos K.T., Kornbluth J. (2016). Cerebral blood flow and autoregulation: Current measurement techniques and prospects for noninvasive optical methods. Neurophotonics.

[34] Rostami E., Engquist H., Enblad P. (2014). Imaging of cerebral blood flow in patients with severe traumatic brain injury in the neurointensive care. Front. Neurol..

[35] Naqvi J., Yap K.H., Ahmad G., Ghosh J. (2013). Transcranial Doppler ultrasound: A review of the physical principles and major applications in critical care. Int. J. Vasc. Med..

[36] Cardim D., Robba C., Bohdanowicz M., Donnelly J., Cabella B., Liu X., Cabeleira M., Smielewski P., Schmidt B., Czosnyka M. (2016). Non-invasive Monitoring of Intracranial Pressure Using Transcranial Doppler Ultrasonography: Is It Possible?. Neurocrit. Care.

[37] Wakerley B.R., Kusuma Y., Yeo L.L., Liang S., Kumar K., Sharma A.K., Sharma V.K. (2015). Usefulness of transcranial Doppler-derived cerebral hemodynamic parameters in the noninvasive assessment of intracranial pressure. J. Neuroimaging.

[38] Robba C., Cardim D., Sekhon M., Budohoski K., Czosnyka M. (2018). Transcranial Doppler: A stethoscope for the brain-neurocritical care use. J. Neurosci. Res..

[39] Bellner J., Romner B., Reinstrup P., Kristiansson K.A., Ryding E., Brandt L. (2004). Transcranial Doppler sonography pulsatility index (PI) reflects intracranial pressure (ICP). Surg. Neurol..

[40] Homburg A.M., Jakobsen M., Enevoldsen E. (1993). Transcranial Doppler recordings in raised intracranial pressure. Acta Neurol. Scand..

[41] de Riva N., Budohoski K.P., Smielewski P., Kasprowicz M., Zweifel C., Steiner L.A., Reinhard M., Fábregas N., Pickard J.D., Czosnyka M. (2012). Transcranial Doppler pulsatility index: What it is and what it isn’t. Neurocrit. Care.

[42] Croal P.L., Leung J., Kosinski P., Shroff M., Odame I., Kassner A. (2017). Assessment of cerebral blood flow with magnetic resonance imaging in children with sickle cell disease: A quantitative comparison with transcranial Doppler ultrasonography. Brain Behav..

[43] Nowaczewska M., Kukulska-Pawluczuk B., Kaźmierczak H., Pawlak-Osińska K. (2019). Post-Lumbar Puncture Headache-Does Hydration before Puncture Prevent Headache and Affect Cerebral Blood Flow?. J. Clin. Med..

[44] Göbel H., Klostermann H., Lindner V., Schenkl S. (1990). Changes in cerebral haemodynamics in cases of post-lumbar puncture headache: A prospective transcranial Doppler ultrasound study. Cephalalgia.

[45] Mowafy S.M.S., Ellatif S.E.A. (2019). Transcranial Doppler role in prediction of post-dural puncture headache in parturients undergoing elective cesarean section: Prospective observational study. J. Anesth..

[46] Chen C.C., Luo C.L., Wang S.J., Chern C.M., Fuh J.L., Lin S.H., Hu H.H. (1999). Colour doppler imaging for diagnosis of intracranial hypotension. Lancet.

[47] Salmon J.H., Timperman A.L. (1971). Effect of intracranial hypotension on cerebral blood flow. J. Neurol. Neurosurg. Psychiatry.

[48] Schmidt E.A., Silva S., Albucher J.F., Luzi A., Loubinoux I., Januel A.C., Cognard C., Payoux P., Chollet F. (2012). Cerebral hemodynamic changes induced by a lumbar puncture in good-grade subarachnoid hemorrhage. Cerebrovasc. Dis. Extra.

[49] Chaves C., Freidberg S.R., Lee G., Zerris V., Ries S., Chavali R. (2005). Cerebral vasospasm following intracranial hypotension caused by cerebrospinal fluid leak from an incidental lumbar durotomy. Case report. J. Neurosurg..

